# Germ-Free Mice Under Two-Layer Textiles Are Fully Protected From Bacteria in Sprayed Microdroplets: A Functional *in vivo* Test Method of Facemask/Filtration Materials

**DOI:** 10.3389/fmed.2020.00504

**Published:** 2020-08-26

**Authors:** Alex Rodriguez-Palacios, Mathew Conger, Fabio Cominelli

**Affiliations:** ^1^Division of Gastroenterology and Liver Diseases, Case Western Reserve University School of Medicine, Cleveland, OH, United States; ^2^Germ-Free and Gut Microbiome Core, Cleveland Digestive Diseases Research Core Center, Case Western Reserve University, Cleveland, OH, United States; ^3^Digestive Health Research Institute, University Hospitals Cleveland Medical Center, Cleveland, OH, United States; ^4^University Hospitals Research and Education Institute, University Hospital Cleveland Medical Center, Cleveland, OH, United States

**Keywords:** COVID-19, respiratory pandemic, cloth masks, fabrics, germ-free mouse model, public droplet safety, coronavirus in schools, decibels speech

## Abstract

Several studies have measured the effectiveness of masks at retaining particles of various sizes *in vitro*. To identify a functional *in vivo* model, herein we used germ-free (GF) mice to test the effectiveness of textiles as filtration material and droplet barriers to complement available *in vitro*-based knowledge. Herein, we report a study conducted *in vivo* with bacteria-carrying microdroplets to determine to what extent household textiles prevent contamination of GF mice in their environment. Using a recently validated spray-simulation method (mimicking a sneeze), herein we first determined that combed-cotton textiles used as two-layer-barriers covering the mouse cages prevented the contamination of all GF animals when sprayed 10–20 bacterial-droplet units/cm^2^. In additional to exposure trials, the model showed that GF mice were again protected by the combed-cotton textile after the acute exposure to 10 times more droplets (20 “spray-sneezes”, ~200 bacterial-droplet units/cm^2^). Overall, two-layer combed-cotton protected 100% of the GF mice from bacteria-carrying droplets (*n* = 20 exposure-events), which was significantly superior compared to 100% mouse contamination without textile coverage or when 95% partly covered (*n* = 18, Fisher-exact, *p* < 0.0001). Of relevance is that two different densities of cotton were equally effective (100%) in preventing contamination regardless of density (120–vs. 200 g/m^2^; *T*-test, *p* = 0.0028), suggesting that similar density materials could prevent droplet contamination. As a practical message, we conducted a speech trial (counting numbers, 1–100) with/without the protection of the same cotton textile used as face cover. The trial illustrated that contamination of surfaces occurs at a rate of >2–6 bacteria-carrying saliva-droplets per word (2.6 droplets/cm^2^, 30 cm) when speaking at 60–70 decibels and that cotton face covers fully prevent bacterial surface contamination.

## Introduction

Since COVID-19 transmits primarily via droplet dispersion from symptomatic and asymptomatic individuals as they talk/cough/sneeze (1), the use of homemade masks is now promoted in most regions for voluntary implementation by the public (2–4). Public compliance, however, varies in part because of a spread of misinformation or disbelief regarding face masks (5). The economic impact of the COVID-19 respiratory syndrome, with doubling times between 2.4 and 5.1 days (6), will disproportionately affect poor communities (7); this is especially true given that the public has limited access to the medical personal protective equipment (including face masks) deemed effective against COVID-19 (8–20).

As an alternative to medical masks, which are in short supply due to the COVID-19 pandemic, our group (21) and others have recently quantified the benefits of textiles (8, 22). Using a spray-simulation method of bacteria-carrying macro and microdroplets, as in rapid *in-vitro* culture methods reported by our group (21) in 2020, reproducibly showed that two layers of cotton textiles were as efficient as medical mask material in reducing the environmental contamination of culture agar surfaces with sprayed droplets. In those spray-simulation studies (mimicking a sneeze), nutritious agar culture media was used to enumerate the number of sprayed microdroplets that could cross the textile.

To complement those studies, the main objective of the present study was to determine to what extent the use of germ-free (GF) mice, in a novel two-layer passive filtration GF housing system [referred to as nested isolation (23)], could be used as a functional model to characterize the benefit of textiles *in-vivo*. We hypothesized that two-layer cotton textiles used as covers could fully protect GF mice from exposure to bacteria contained in microdroplets sprayed on the other side of the textile. The goal was to quantify the potential for absolute prevention of micro-droplet dissemination into the textile-covered GF mouse cage/environment (binary data, yes/no GF mouse contamination). For the first time, GF animals are proposed as an effective *in vivo* system to assess microbial sterility, as a functional test of textiles for use as face masks and surface covers, furthering gathering data to promote a “Universal droplet reduction model” to control rapid respiratory pandemics. We also explored this further with a trial of droplet production/contamination during speech.

## Methods

Herein, we conducted studies using laboratory GF Swiss Webster mice to determine how effective household textiles are as barriers to protect the mouse environment against contamination by a mixture of bacteria-containing microdroplets using a spray simulation method (21).

### Textiles

From a series of textiles recently tested in our laboratory (21), we selected 100% combed cotton (a widely available, “T-shirt material”); fabric density clustered around two types, 120 and 200 g/m^2^ (GSM). This material was selected because two-layer cotton textiles were one of the most effective options at retaining sprayed liquid droplets containing bacteria during culture-based *in vitro* testing, as we demonstrated early in 2020 (21). Textiles were wrapped using surgical strategies as for surgical drape preparation, individually wrapped in ink-free paper, and autoclaved prior to use. At the time of use, the two layers were manually separated to eliminate the areas where heat had “glued” the two layers as one. Handling of materials was conducted strict aseptic measures as they are customary and previously described in our GF research facility (23).

### Animals and Germ-Free Facility

The *in vivo* testing of such materials for the present study were conducted using GF Swiss Webster mice available from our Germ-Free and Gut Microbiome Core facility. The mouse line was obtained originally obtained from Taconic Biosciences Inc. (Hudson, NY). Animals were maintained using a portable static isolation strategy widely validated in our laboratory (23). Verbatim (24), as previously described in detail (23, 25), mice were maintained as GF colonies at the Animal Resource Center at Case Western Reserve University (CWRU) School of Medicine. Animals were housed in wire-topped polycarbonate shoebox cages (~30 cm L; 15 cm W; 15 cm H) in a 12 h:12 h light:dark cycle. Autoclaved GF-grade 40–50 kGy irradiated pellet food (PMI Nutrition Int'l., LLC., Labdiet® Charles River. Vac-Pac Rodent 6/5 irradiated, 5% kcal% fat) diets and water in bottles were provided *ad libitum*. Protocols on animal handling, study designs, and housing were approved by the IACUC at CWRU in accordance with the National Research Council Guide for the Care and Use of Laboratory Animals (26). To promote rigor and analytical reproducibility (24), GF animals were individually caged, eliminating the need to control for cyclical bias (23) or cage-clustered data (27).

### Bacterial Solution

Since respiratory viruses exist in association with bacteria in respiratory fluids (28, 29), we used a bacterial-suspension spray simulation method (previously described) to quantify the number of droplets that could not be visualized but that could escape textile barriers, as recently validated by our group. In brief, we used a bacteria-carrying microdroplets spray simulation method where spray bottles were filled with an aqueous suspension of 12-probiotic-cultured dairy product (*Lactobacillus lactis, L. rhamnosus, L. plantarum, L. casei, L. acidophilus, Leuconostoc cremoris, Bifidobacterium longum, B. breve, B. lactis, Streptococcus diacetylactis*, and *Saccharomyces florentinus*, 75 ml; 3 × 10^6−7^ cfu/ml, 25 ml Saliva 10^6−7^) in 200 ml PBS (Fisher BP-399-1) to simulate a cloud of droplets produced by a sneeze (21). Probiotics are BSL-1/ “Generally Recognized As Safe” by the FDA and all experiments were conducted in BSL-2 HEPA-filtered microbiology laboratories. No human subjects were used for experimentation. The parallel lanes plating method was used to enumerate the bacterial counts in final solution (30).

### Spray Simulation

Before testing, spray bottle nozzles were adjusted to produce cloud and jet-propelled droplets that match the visual architecture of droplet formation described by Bourouiba et al. (28). Specifically, we used a high-volume trigger single-v-orifice nozzle sprayer (1.0 ml per stroke) with 28/400 neck and 9-1/4-inch dip tube fitted with a filter screen (model PA-HDTS-EA, Mfr. Model # 922HL, Delta Industries, Inc.). Before conducting the experiment with animals, infrared imaging technology was used to illustrate that the spray model was composed of various liquid phases occurring within a single spray (1 ml/stroke), revealing a wide arrange of droplet sizes (right skewed distribution ranges between 20 and 900 micrometers with a peak at 70–100 micrometer) (31). In context, the size of droplets in the human sneeze ranges between 40 and 900 micrometers, with most droplets (70–100%) normally or bimodally distributed around 360–390 micrometers (32). The spray bottle ejects fluid with pressures that can reach 10 psi—sufficient to create a short burst of fluid/jet and fan cloud. In perspective, the pressure during a sneeze is between 1 psi (51.7- mmHg) in the trachea, and 2.6 psi in mouth/pharynx (135 mmHg), which can be reached in 0.1 s (33), while exhalation during strenuous activity reaches tracheal pressures of 0.03 psi (1.55 mmHg).

### Droplet Quantification

To quantify the droplet exposure per surface area we used 10-mm-Petri dishes containing tryptic soy agar (56.75 cm^2^ surface area/dish) with 5% defibrinated sheep blood placed on the center of cages. Cages and the agar remained covered or open for 10 min following spray bottle droplet dispersion to allow droplet landing. Before conducting the experiment with animals, infrared imaging technology was used to visually illustrate that the spray model using the methods described earlier by our group, and a liquid suspension at 46°C, on a background set at 21°C (23, 34).

### Gf Housing System

Animal experiments were conducted with a system of germ-free-grade nested isolation (23) where a cage of a smaller size is nested into another one of larger capacity, each containing their respective Remay passive filtration filter as a lid for a total of two layers of filtration. In this study, the two layers of Remay filters (the cage lids) were replaced by two layers of 100% cotton material. Upon replacement of the lid's material, sets of cages (500 cm^2^ floor area/cage) with individually caged (litter mate) GF mice were sprayed with the bacterial suspension, covered, or uncovered with the textiles at various distances and spray doses, 10 cm above the lid cage plane. For clarity, the “no-textile barrier controls” were the cages that remained open without a lid. Thirty seconds following the spray of the droplet-cloud, textiles were removed, and the two Remay filter lids were placed back on the nested cages.

### Repeated Droplet Exposure of Mice

**Figure 1:**
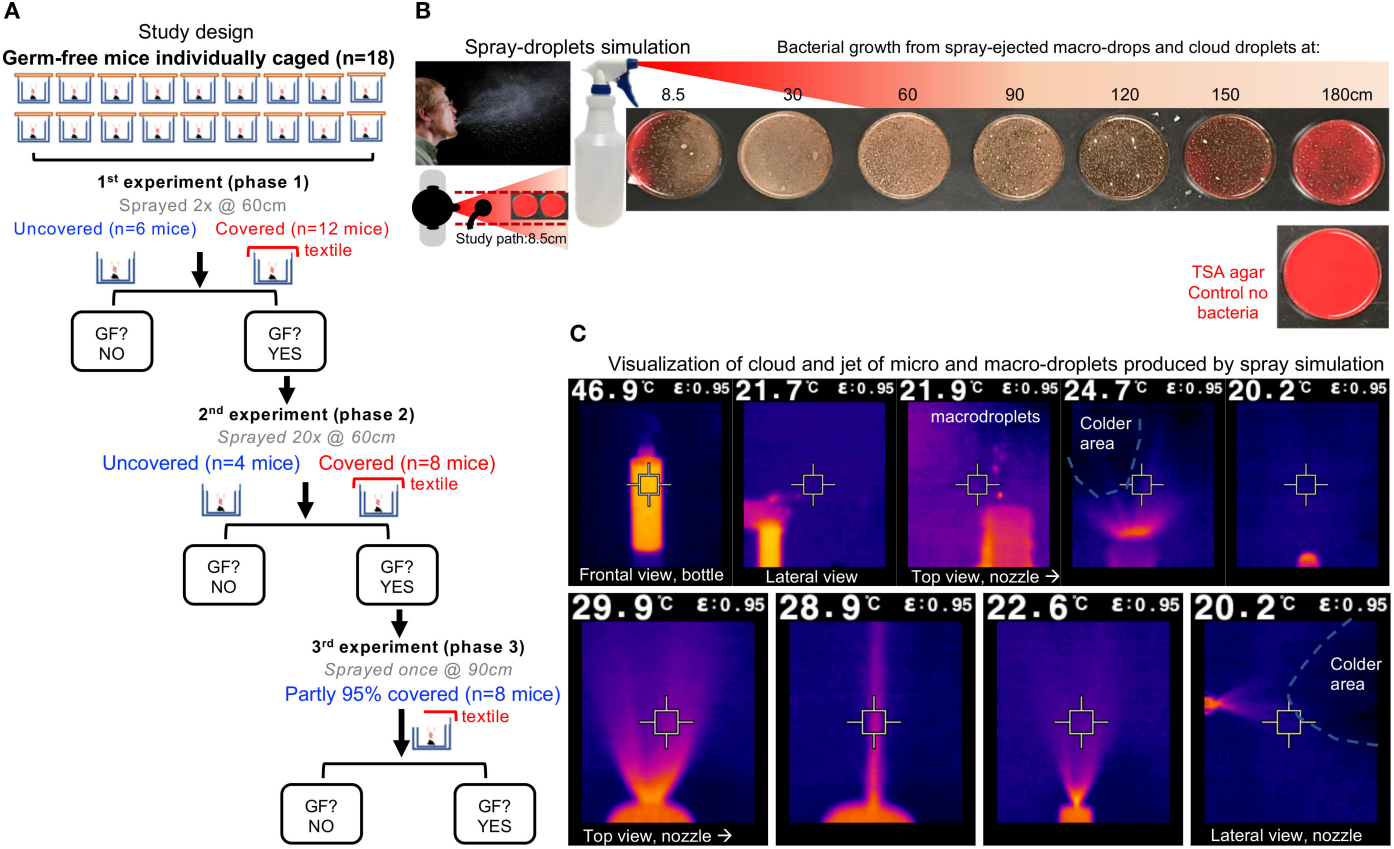
Study overview and infrared imaging of ejection features of spray model. **(A)** Study overview. The experiment phases were conducted at different distances and with different droplet exposure doses. One mouse/cage. Each spray trail was counted as a droplet exposure event. Outcome, GF contamination 5 d after spray: yes/no. “GF?,” question on whether mice remained GF, when tested after 120 h post-spray exposure to droplets, to select GF mice and continue with re-exposure experiments. If “no,” end of study for those mice. If “yes,” animals were GF and re-exposed to droplets. **(B)** Spray-droplet simulation model using bacterial solution as recently validated by our group for the assessment of textiles; unmodified from Rodriguez-Palacios et al. (21); open access Creative Commons Attribution (CC BY) (http://creativecommons.org/licenses/by/4.0/). Note the TSA agar plate shown has no bacteria (control no bacteria), and notice the red color of the agar changing to brown as increasing number of whitish dots (bacterial carrying microdroplets) land on the agar surface. **(C)** Infrared features of cloud-droplet ejection. Infrared imaging is based on infrared light which is electromagnetic radiation with long wavelengths that are invisible to the naked eye. Being a form of radiation, heated objects (the solution in the spray bottle) emit infrared light which contrast the lower background temperature in the room (blue or black). Electromagnetic waves carrying radiant heat energy from objects that loose heat/energy is detected by the camera, which is shown. For imaging the spray bottle was filled with liquid solution at 46.9°C (vs. Room temp of 20.2°C, humidity 70%); see high-volume trigger sprayer details in methods. The square in the center of the picture matches the color inside the square, and the temperature is shown at the top of the image. Close-up pictures illustrate the fluid ejection in proximity to the spray bottle nozzle. Notice that the solution rapidly cools down upon ejection as spray (shown as black colder area effects). Note that the simulation model resembles the features of sneeze fluid dynamics (28), with wide dispersion of high-velocity microdroplets, large heavy macro-droplets, a long-range projectile-like jet, and a large conical cloud.

In short, the droplet exposure experiment was conducted in three phases (see flow chart of study overview/design in Figure 1A). In the **first phase**, 18 GF Swiss Webster mice (males:females, 1:1) aged 9 weeks were individually caged in our GF-grade NestIso caging system. Mice were assigned to two groups, 12 “textile-cover” and six “no-cover.” Lids were temporarily removed from all cages for the spray simulation test. Twelve mouse cages were covered with the two-layer textile (“textile cover” group) while the remaining cages remained uncovered (“no cover;” no lid and no textile barrier). Each cage was then sprayed twice (spray nozzle was located at 60 cm from the cage). To determine if the droplet cloud had crossed the textile barrier, contaminating the GF environment and causing the colonization of animals, fecal samples of all animals were collected aseptically from each animal, 36 and 120 h after droplet exposure. Upon confirmation of the GF status at 120 h (5 days, end of phase one) all mice that remained GF at 120 h were then used for the **second phase** of the experiment: repeated exposure a cloud of sprayed microdroplets. Using the same strategy (covered vs. uncovered paired side-by-side cages), two thirds of the GF mice were exposed to 20 sprays (instead oftwo2; 10 times more droplets) at 60 cm, while the remaining third were left uncovered and sprayed only once at 180 cm (relevant to uncovered individuals at the recommended social distance). Feces were again measured at 36 and 120 h. Upon conformation of mouse GF status after 120 h (end of phase two), we then conducted the **third phase** experiment. Using all the mice that remained GF from phase two, phase three was conducting by covering only 95% of the cage with the two-layer textile (“partly covered” group, cages were covered, except for a corner of 5% of cage area). In this experiment, all cages were sprayed once at 90 cm. Culture of feces for confirmation of GF status was verified 120 h later.

### Droplet Production During Human Speech

To put the spray experiments with GF mice into practical perspective for humans, we demonstrated the effectiveness of the cotton textile in retaining/reducing the risk of environmental contamination by oral/saliva droplets produced by one of the investigators (a healthy volunteer) during a speech trial (counting from 1 to 100 in English) conducted at 30 cm over a sterile TSA (Becton Dickinson) agar plate. Speech intensity and background noise in decibels were measured with The National Institute for Occupational Safety and Health (NIOSH) Sound Level Meter (SLM) phone app, which was placed at 90 cm (arms' reach) from the mouth. The app was developed to help individuals monitor their noise environment and promote better hearing health with accuracy of ±2 decibels. The app is freely available at app stores and from the Centers for Diseases Control and Prevention website https://www.cdc.gov/niosh/topics/noise/app.html. The speech trial, conducted by the lead investigator (healthy individual), is not considered human experimentation or subject research.

### Statistical Analysis

Each time a GF mouse was exposed to a spray simulation, the event was deemed independent and referred quantitatively for binary data count statistics to as a “GF mouse exposure event.” Colonization data was compared between fully covered and non- or partly-covered cages using Fisher's exact test and STATA. *Post-hoc* study power statistics were computed for each analysis as recently described by our group (24). To promote open access and review, this manuscript was made available as a preprint for community contribution upon submission for peer-review (34). Sample size estimations using the open-access software G^*^power (24), for expected 0.1 vs. 99.9% colonization:protection, for two samples at a n1:n2 ratio of 1:1, and one-tailed *P* < 0.05, revealed that five mice per group was sufficient to achieve a power of 0.99. Since the main outcome was the presence or absence of sterility (or the permanence of GF status), the binary status (yes/no) data were analyzed using Fisher's exact test (n exposed/n contaminated by droplets) to determine if the shirt material density was a factor determining the risk of droplet retention failure (STATA, v15.1). Confidence intervals (95%) provided convey information relevant to sample size. Textile density GSM (grams /squared meter) was tested using unpaired *T*-test with Welch correction for unequal variances. Paired *T*-tests were used for textile imaging and ImageJ data analysis.

## Results

Infrared imaging technology illustrating the various liquid phases occurring with our spray simulation model, revealed a wide arrange of droplet sizes and velocities, thus demonstrating that the mouse cages were exposed to a fast-moving jet and cloud of macro and microdroplets, mimicking a sneeze (Figures 1B,C).

**Figure 2:**
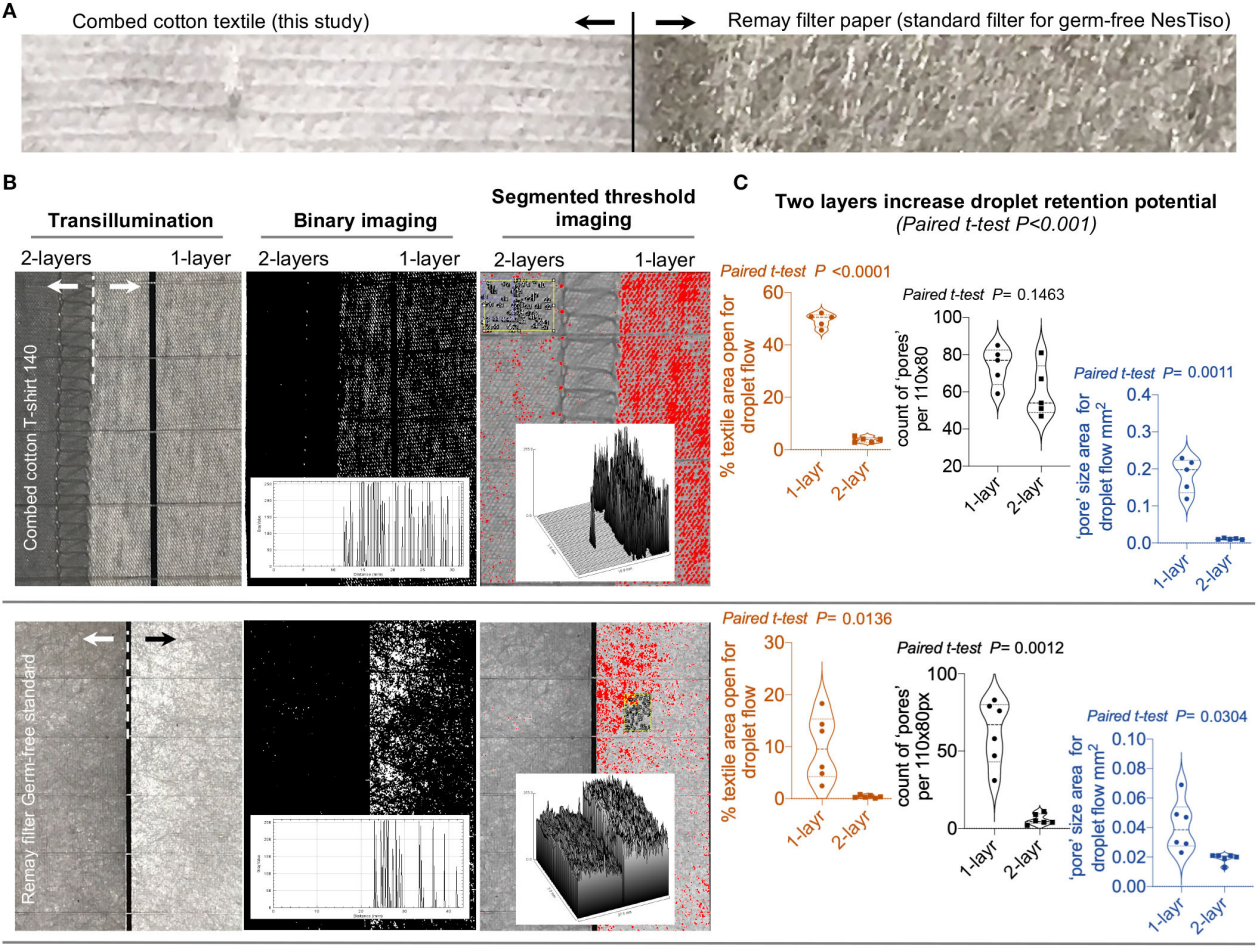
Image analysis with ImageJ to characterize the white light transillumination porosity of 2-layer textiles. **(A)** Photograph of the cotton textile tested with GF mice in this study compared to a Remay^TM^ filter-sheet (Spunbonded Polyester Non-woven Fabrics) used as an analytical “gold-standard” in cage lids for Nested isolation (23) as comparator. **(B)** ImageJ analysis of single and double layers of both cotton and Remay materials. Histogram and surface plots illustrate significant reduction of light passage through textile pores; notice two layers. **(C)** Statistical features of double layers promote increased retention of droplets (pore counts/areas size).

Trans-illumination and ImageJ analysis of the textile material (23, 34) used for covering the mouse cages and protect the GF mice from sprayed droplets, revealed a profound reduction (up to 10-fold) of individual and total “pore” area (from 50% of textile area as single layer to 5% as two layers) and counts that allow the flow of light for the cotton textile compared to the “gold-standard” GF-grade Remay filter (Figures 2A–C).

Textile data supported the use of two-layer textile barriers for the *in vivo* experiments. In the first phase of the spray experiment with mice, microbiological analysis (fecal culture) of mouse feces before and after two rounds of spray-droplet exposure (2 ml total) at an inoculation dose of 600–1,000 bacterial droplet units per 56.75 cm^2^ showed that all GF animals with no textile protection (simulating not wearing a mask) showed signs of microbial contamination within 36 h. In contrast, the GF status of the mice that were covered with the autoclaved textile remained GF after exposure (measured at 120 h), indicating that the textile barrier was extremely effective at retaining bacteria carrying droplets, thus reducing the absolute contamination risk (0/12 vs. 6/6, Fisher's exact, *p* < 0.0001).

The second phase of the experiment testing repeated spray exposure (20 sprays; 10 times as many droplets that initial phase experiment, 20 ml volume of liquid per mouse cage), with 12 GF mice, showed that the textile maintained all animals GF, even after 20 droplet sprays at 60 cm, while mice located at 180 cm became colonized by bacteria-carrying droplets with a single spray (0/8 vs. 4/4, Fisher's exact, *p* = 0.002). Collectively, barriers protected all mice (even with low textile density; heavy vs. light fabric, paired *t*-test, *p* = 0.002) against high droplet doses two or 20 sprays) if the textile fully covered the cage (0/20 vs. 10/10, Fisher's exact, *p* < 0.0001, study power = 1.0).

**Figure 3:**
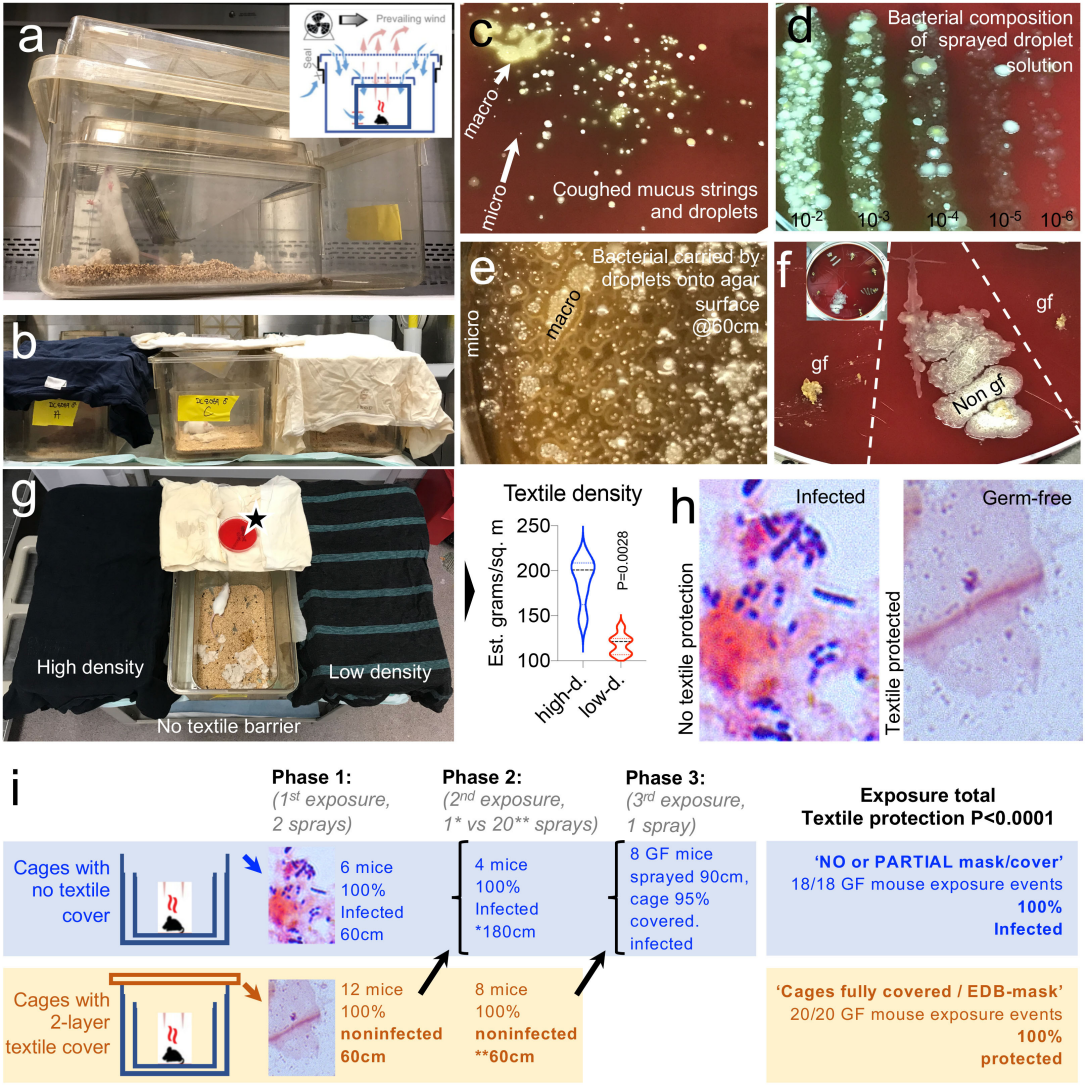
A two-layer textile barrier fully protects germ-free mice from colonization by bacteria in sprayed microdroplets. **(a)** Nested isolation cage housing two-layer system used to raise GF mice (23). **(b)** In this experiment, the two cage lids were replaced by a two-layer textile barrier cover compared with cages without a lid (no cover). Sprayed from 60 to 180 cm distances (see Methods). **(c)** Visualization of bacteria present in cough microdroplets of a healthy adult volunteer. TSA plates, aerobic incubation, 48 h. Note the color, number, size, and relative location and distribution of the bacteria colonies growing from “invisible” microdroplets (CFU) shown as whitish spots on the agar surface. Bacterial growth alters the red color of the fresh non-inoculated agar leading to a brownish discoloring of the petri agars, which is more pronounced as the number of bacterial colonies increase. **(d)** Quantification/visualization of bacterial community in microdroplet solution used to spray GF mice. Parallel lanes plating method (30). **(e)** Visualization of bacteria-contained on macro/microdroplets sprayed on TSA. 21 mm horizontal field. **(f)** Example of fecal culture-negative from mice protected with textiles, which remained GF (gf), and culture-positive from mice not protected with textile (Non-gf), Inset, 20 cm plate, eight samples. **(g)** Two textile densities were tested, but both protected gf mice. Notice the uncovered cage at the center with an open TSA plate located over the cover to verify and quantify the bacteria-carrying microdroplet density that the mice were exposed to. **(h)** Feces, gram stain. See details in Supplementary Figure 1. **(i)** Summary of *in vivo* mouse droplet exposure event results. Refer to overview of study design in Figure 1A as a referent.

In the last phase of the spray-experiment, partly covered (95%) cages revealed that, compared to fully unprotected cages, one single dose of droplets at 90 cm of distance (1-spray, ~0.2–0.6 × 10^3^ microdroplets) resulted in the bacterial colonization of all (*n* = 8) mice. Collectively, the number of GF mice that remained GF with a cage fully covered was significantly superior (0/20) compared to the number of mice that were colonized in non-covered or partly covered cages (18/18, Fisher's exact *p* = 1.14E-06, study power = 1.0; Figures 3a–i).

**Figure 4:**
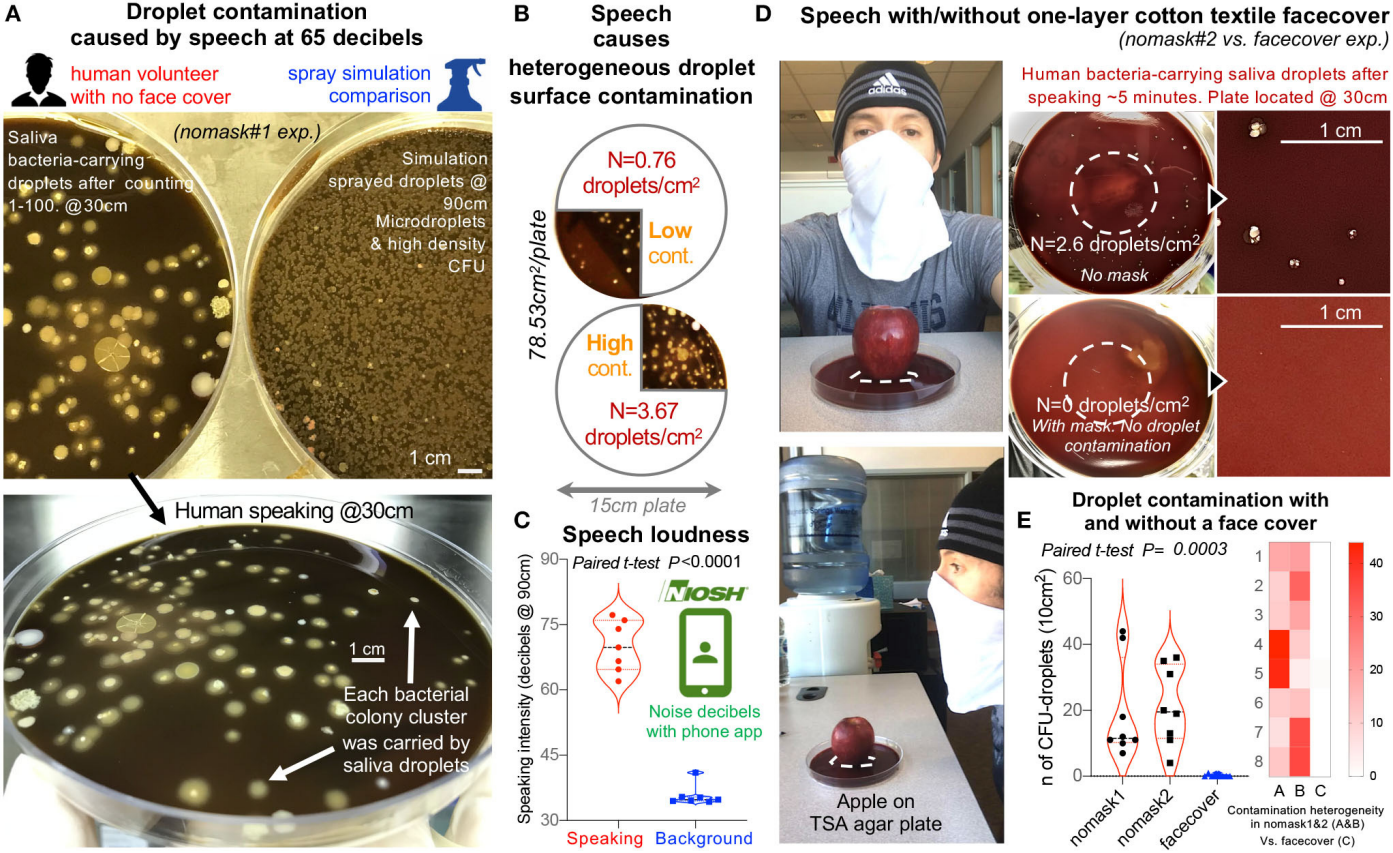
Textile cotton face masks effectively prevent the contamination of the environment with microbial-carrying saliva microdroplets produced by a human volunteer during speech. **(A)** Comparison of bacterial CFU density of plates contaminated by oral droplets during speech (counting from 0 to 100, taking breath every 15–20 numbers, at 30 cm distance from plate) vs. plates with sprayed microdroplets at 90 cm. Large TSA agar plates, 150 mm diameter, and aerobic incubation at 72 h, 37°C. **(B)** Speaking without a face cover causes heterogeneous contamination of environmental surfaces. **(C)** Speech intensity and background noise in decibels (60–75 decibels during speech trials nomask#1 on **(A)** and #2 vs. face cover in **(D)**. Background noise and speech volume intensity were measured in decibels using the Centers for Disease Control and Prevention free phone application NIOSH Sound Level Meter App (https://www.cdc.gov/niosh/topics/noise/app.html). **(D)** Speech experiment repeat by human volunteer with and without a face cover as illustrated, quantified droplet contamination density of between 0.5 and 4 droplets/cm^2^ after counting numbers from 1 to 100, and prevention of surface contamination using a single textile layer/barrier. The apple on the agar is to provide a visual and spatial context of the relevance of face covering during speech over food displays/service settings, and to provide the context for a citizen science project we have proposed for adults and children to use the spray simulation model to test the efficacy of face coverings and numerous household textiles available in their homes (see links to websites and educational modules in Spanish, French, English, and Portuguese in Eichler et al., in ref (35). The apple was washed and clean with cloth and ethanol 70% before placed on agar. The dashed lines show area where apple was placed. Note that clean apple yielded no bacteria contamination on agar). **(E)** Summary of droplet contamination for the second speech trial illustrate that, while face covers prevent surface contamination (e.g., 2–6 saliva droplets/cm^2^ @30 cm; >2–6 droplets per word based on 78.53 cm^2^ plate), the lack of face masks renders the environment heterogeneously contaminated with droplets.

To put the spray experiment in GF mice in human context and perspective, we then tested the ability of the same two-layer cotton textile barrier, used as a face cover, to prevent environmental contamination of an agar surface 10 cm in diameter located at 30 cm with droplets during speech (counting numbers out loud from 1 to 100) conducted within 60–75 decibels. The lack of droplet protection during speech causes the contamination of the environment with bacteria-carrying oral droplets, at heterogeneous densities ranging from 0 to 5 droplets/cm^2^ after the short speech trials when measured at 30 cm of distance from the lips. Figures 4A–E illustrates that even a single layer of the material used in the experiments above, as spray simulated in another study, was effective at retaining/reducing the risk of environmental contamination by oral/saliva droplets compared to not using a face cover.

## Discussion

This study illustrates that GF animals could be used as a functional *in vivo* model to test the effectiveness of textiles as droplet barriers. When protected by two layers of textile (100% combed cotton), all mice were 100% protected from becoming contaminated by the bacteria contained in the microdroplets. In this context, the study supports that the use of textiles as face covers could be an effective prevention strategy to halt the contamination of the environment with respiratory and saliva/oral microdroplets which may contain known and unknown infectious microorganisms (36, 37).

Although inspired by the current COVID-19 situation and our working model to promote textile face masks and surface covers (21), this study was not intended to address the complex biology of viral infections in humans or as a means to replace long-validated N95 masks, which are fit-tested directly in humans (8). Rather, our study sought to test, *in vivo*, whether two-layer textiles would be effective at preventing the crossing of liquid droplets, mimicking a sneeze. To put the findings into context, our speech trial illustrated that human speech is a constant source of droplet production and contamination. Most importantly, the speech trial illustrated that the textiles tested herein prevented the contamination of the environment with saliva borne microorganisms.

This is the first available study of its kind using GF mice to assess the functional filtration efficiency of liquid micro-droplet material amenable for the fabrication of face masks or covers. Following the pre-print publication of the present study (34), a widely publicized, yet unpublished, study with hamsters indicated that “masks” reduced the contamination of animals with COVID-19 virus by 75% when animals were confined within cages for a week (38). Such preliminary report supports the importance, effectiveness, and value of using surrogate *in vivo* models to study droplets and masks. In future pandemics, the limited access to viruses, or the unwanted need to use such viruses to study face mask effectiveness could be early initiated before the pandemic accelerates using models based on bacterial carrying microdroplets. Toward the future, animal models could be used to further examine the role of droplet barriers in preventing the respiratory transmission of viral particles, for instance, the murine hepatitis *Coronaviridae* virus (39). Although we assessed combed cotton textiles of two densities, studies indicate that most textiles would be effective (21), and beneficial for the control of viral particles (40), or nanoparticles especially if cotton and electrostatic materials are used as a combination in cloth face masks (10, 22).

Of remarkable interest to animal and biomedical research, the textiles herein tested, using the GF-based testing model and NesTiso, were unexpectedly 100% effective at preventing contamination of the mice with the liquid microdroplets. These findings are remarkable because they further support our earlier work in 2018 where we proposed a novel system of breeding and isolation of GF animals using non-pressurized HEPA-filtration anchored methods based on two-layer “nested” isolation (NestIso, nested isolation) (23). In that study, serology conducted at 62 weeks in mice demonstrated that all animals had no titers against 18 highly contagious rodent viruses, including betacoronavirus [see Supplementary Materials in (23)]. Together, findings support the potential to rapidly expanding the research capabilities of using Nested isolation to promote the use of GF animals in disease/microbiological research and assist microbiome research reproducibility (24, 27).

### Limitations and Future Directions

The science of textiles is complex, and the study of textiles in particulate/air filtration using *in vitro* systems is becoming a re-emerging field of research since the occurrence of increasingly devastating respiratory pandemics, especially COVID-19 (10, 21, 22, 40). As a novelty, our study was designed to effectively illustrate, as a proof-of-principle, the use of our germ-free mouse housing system/model to examine the filtration potential of any type of materials in an innovative *in vivo* animal system. As such, our findings on the textile specifically used to illustrate the GF model cannot be generalizable to other types of filtration materials, or the number of layers, since each material has their own porosity and hypothetical ability to retain dry and wet droplets or particulates. Future studies could study combinations of materials, practices, or animal genetic lines, or features of the gut microbiota that could modify the susceptibility to droplet-driven infections to tailor current and new potential questions across various fields of science.

Projecting the message from this report into the future via education, along messages from an earlier study from our group on the role of textile barriers reducing droplet contamination distances21, the present studies were used to further support strategies and the need to publicize the relevance of facemasks in the community, especially in schools, as students and workers start returning to highly-populated classrooms and institutions. To promote such efforts, this and our preceding complementary study21 have been used as the foundation to create educational research activities amenable for children and adults, at school and at home, and a citizen science facemask experiment project concurrently launched in multiple languages (Spanish, French. Portuguese and English) to promote COVID-19/coronavirus safety and droplet science awareness (35).

In conclusion, the GF animal protocol herein described is a rapid reliable functional *in vivo* model to test the effectiveness of textiles as droplet barriers or other filtration materials required for infection control or high sterility purposes. Together, the mouse experiment and the speech trial emphasize the benefits of using textiles to enhance the cleanliness of the environment, which can be contaminated by oral-respiratory droplets, regardless of which natural or infectious microorganisms are contained within the droplets.

## Data Availability Statement

The raw data supporting the conclusions of this article will be made available by the authors, without undue reservation.

## Ethics Statement

The animal study was reviewed and approved by IACUC Case Western Reserve University.

## Author Contributions

AR-P envisioned, planned, executed the experiments, analyzed the data, prepared figures, and wrote the manuscript. MC assisted with experiments. FC commented, revised, and edited the manuscript. All authors approved the final manuscript.

## Conflict of Interest

The authors declare that the research was conducted in the absence of any commercial or financial relationships that could be construed as a potential conflict of interest.
