# Supplementary material for: Germ-Free Mice Under Two-Layer Textiles Are Fully Protected From Bacteria in Sprayed Microdroplets: A Functional in vivo Test Method of Facemask/Filtration Materials
Source: Front Med (Lausanne). 2020 Aug 26;7:504. doi: 10.3389/fmed.2020.00504 (PMC7479817; doi:10.3389/fmed.2020.00504)
Supplement: Supplementary file 1 [file Data_Sheet_1.docx]

**Germ-free Mice Under Two-Layer Textiles are Fully Protected from Bacteria in Sprayed Microdroplets: A Functional *in vivo* Test Method of Facemask/Filtration Materials**

Alex Rodriguez-Palacios, DVM PhD ^1,2^* Mathew Conger, BSc^1^, Fabio Cominelli, MD PhD ^1,2^

^1^Division of Gastroenterology & Liver Diseases, Case Western Reserve University School of Medicine and ^2^Digestive Health Research Institute, University Hospitals Cleveland Medical Center, Cleveland, OH, USA.

***Corresponding Author:** Alex Rodriguez-Palacios ([axr503@case.edu](mailto:axr503@case.edu))

**Supplementary Figure 1**

**
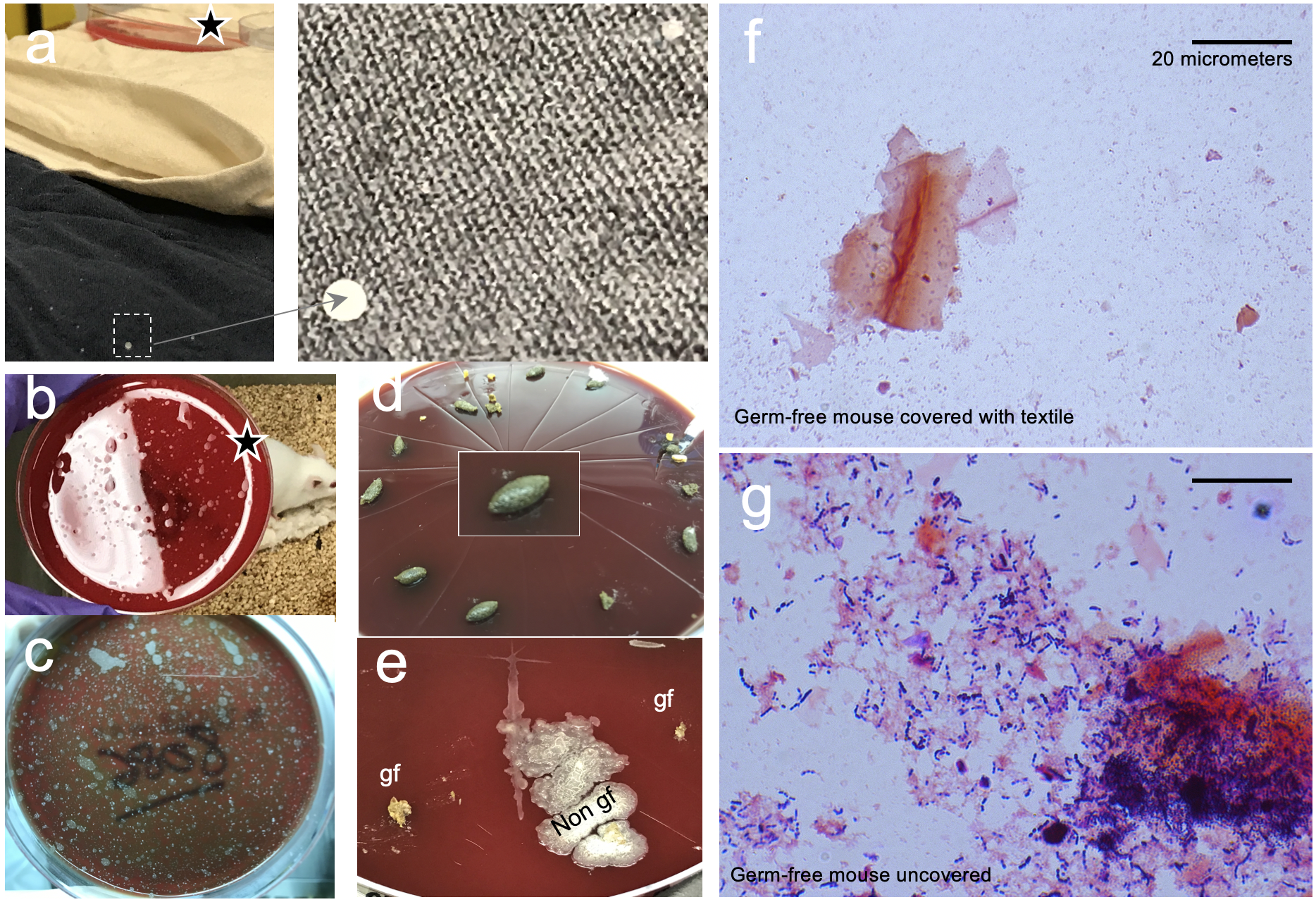
Supplementary Figure 1.** **Textile Droplet Barrier fully protect germ-free mice from microbial colonization by bacteria present in sprayed liquid micro-droplets**. **a**) Textiles were able to retail large drops and microdroplets. **b**) Agar plate shows droplet density to which mice were exposed immediately after spray (stars). **c**) Aerobic incubation of agar illustrates environmental contamination of mouse cage with numerous microdroplets not visualized immediately after spray. **d**) Fecal samples from all mice show no bacterial growth after 36h of incubation on agar before experiment confirming Germ-free status of mice. **e**) Fecal samples from representative mice protected with textile showing no bacterial growth after 36h of incubation on agar after spray confirming Germ-free status protection by the textile, and no-barrier mice showing fecal bacterial growth. **f-g**) Representative gram stain of fecal samples in this study shown as insets in the manuscript**.**

**Supplementary References.**

1 Bourouiba, L. IMAGES IN CLINICAL MEDICINE. A Sneeze. *N Engl J Med* **375**, e15, doi:10.1056/NEJMicm1501197 (2016).

2 J, A. *et al.* Natural Ventilation for Infection Control in Health-Care Settings. Annex C Respiratory droplets. Geneva: World Health Organization; 2009. Bookshelf ID: NBK143281. (2009).

3 Rodriguez-Palacios, A. *et al.* 'Cyclical Bias' in Microbiome Research Revealed by A Portable Germ-Free Housing System Using Nested Isolation. *Sci Rep* **8**, 3801, doi:10.1038/s41598-018-20742-1 (2018).
